# Intercalary fragments in posterior malleolar fractures: incidence, treatment implications, and distribution within CT-based classification systems

**DOI:** 10.1007/s00068-022-02119-2

**Published:** 2022-11-04

**Authors:** Elena Mueller, Holger Kleinertz, Marlon Tessarzyk, Stefan Rammelt, Jan Bartoníček, Karl-Heinz Frosch, Alexej Barg, Carsten Schlickewei

**Affiliations:** 1 Department of Trauma and Orthopaedic Surgery, University Medical Center Hamburg-Eppendorf, Martinistr. 52, 20246 Hamburg, Germany; 2 University Center of Orthopaedics, Trauma and Plastic Surgery, University Hospital Carl Gustav Carus at TU Dresden, Dresden, Germany; 3 Department of Orthopaedics, First Faculty of Medicine, Charles University, Central Military Hospital Prague, Prague, Czech Republic; 4 Department of Trauma Surgery, Orthopaedics, and Sports Traumatology, BG Hospital Hamburg, Hamburg, Germany

**Keywords:** Intercalary fragment, Posterior malleolar fracture, Ankle fracture, Bartoníček/Rammelt

## Abstract

**Introduction:**

Complex ankle fractures frequently include the posterior malleolus (PM). Despite advances in diagnostic and treatment strategies, PM fracture involvement still predisposes to worse outcomes. While not incorporated into the most common PM fracture classifications, the presence of an intercalary fragment (ICF) complicates treatment. This study aims to describe the incidence, morphology, and location of ICFs in PM fractures.

**Materials and methods:**

A total of 135 patients with a mean age of 54.4 (SD ± 18.9) years and PM fractures were analyzed for the presence of an ICF. Patients with an ICF were compared to those without in terms of age, gender, and treatment received. Characteristics of the ICFs in terms of location and size were assessed. Furthermore, the presence of an ICF in relation to the PM fracture classification according to Haraguchi et al., Bartoníček/Rammelt et al., and Mason et al. was investigated.

**Results:**

ICFs presented in 55 (41%) of the 135 patients. Patients with an ICF were younger, and the PM was more often operatively treated when compared to patients without an ICF. A posterolateral approach was used significantly more often in patients with an ICF. Almost all ICFs were found in the posterolateral (58%) and posterocentral (35%) regions. The majority of fragments were found in Bartoníček/Rammelt type 2 fractures, the most common fracture type. Bartoníček/Rammelt type 3 fractures had the highest relative frequency of ICFs.

**Conclusion:**

ICFs are frequently found in PM fractures; however, they are not incorporated into any of the common classifications. They are generally found in younger patients and associated with more complex PM fractures. As they can complicate reduction of the main fragment and may require direct exposure to restore joint congruency, ICFs should be considered in PM fracture classifications. Due to their location, the majority of ICFs are able to be accessed using a posterolateral approach.

## Introduction

Ankle fractures account for 4–9% of all fractures [1–5]. The posterior malleolus (PM) is involved in up to 50% of ankle fractures [6–16]. Despite increasing knowledge about the three-dimensional PM fracture pathoanatomy, PM fractures still predispose to poorer functional outcomes when compared to other ankle injuries [8, 10, 17]. Some controversy still exists about how to correctly treat complex fractures involving the PM [8].

The use of a computed tomography (CT) scan, currently the gold standard in the diagnosis of trimalleolar fractures, allows for a better understanding of fracture characteristics including geometry, comminution, impaction, dislocation, as well as the number and location of fragments. CT-based findings in combination with biomechanical studies replaced old treatment rules, including the 1/3 rule by Nelson and Jenson [18]. Furthermore, several study groups have developed CT-based classifications, with the most commonly used by Haraguchi et al. [19], Bartoníček/Rammelt et al. [20], and Mason et al. [21]. Despite good inter- and intraobserver reliability, all of the mentioned classifications have pitfalls and do not consider certain relevant aspects of treatment, such as comminution or the presence of intercalary fragments (ICF) [15]. In particular, dislocated ICFs trapped within the fracture frequently change the treatment plan and choice of surgical approach [12, 22, 23].

The aims of this retrospective study were: (1) to determine the incidence of ICFs in PM fractures and if they have treatment implications; (2) to analyze the morphology, location, and distribution of ICFs within the three established CT-based classifications by Haraguchi, Bartoníček/Rammelt, and Mason.

## Materials and methods

### Study population

The present study was conducted at a level I trauma center in the second largest city in Germany. Patients with complex ankle fractures involving the PM who received operative treatment and obtained a preoperative CT scan were included in the study. Ankle fractures without PM involvement, and tibial pilon fractures were excluded. Further exclusion criteria included age under 18 years, history of prior ankle injury, infection, and Charcot arthropathy. A total of 135 patients could be included between October 2018 and September 2021. Patient information and treatment history were gathered from electronic medical records by two independent observers.

The study was approved by the local ethics committee (WF-093/21) and conducted in accordance with the ethical standards laid out by the 1964 Declaration of Helsinki.

### Intercalary fragment analysis

**Fig. 1:**
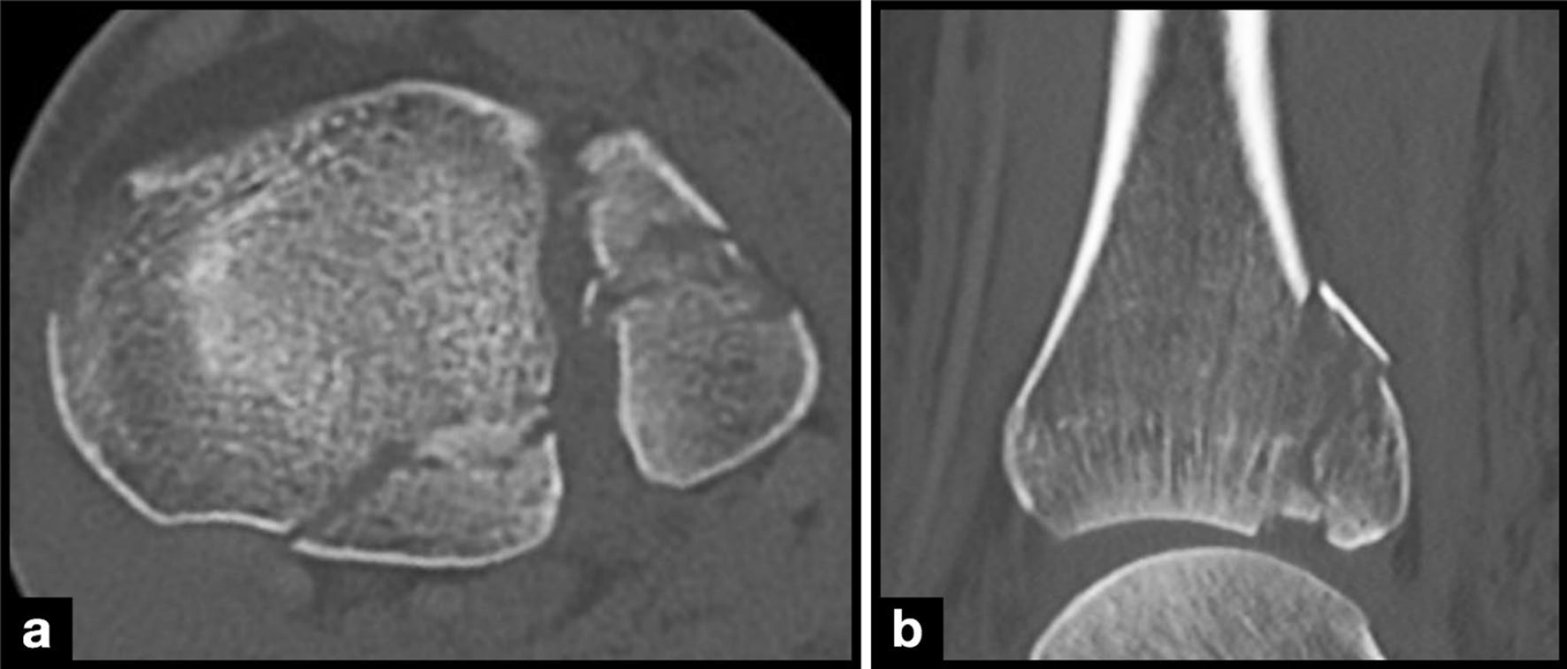
CT scan with a dislocated intercalary fragment in the **a** axial and **b** sagittal plane

We analyzed the prevalence, location, dislocation, size, approximate volume, and distribution of ICFs within the three above mentioned CT-based classifications. The ICF was considered dislocated when there was displacement from the articular surface (Fig. 1). All CT scans were anonymized and saved as Digital Imaging and Communications in Medicine (DICOM) files. Four independent observers (levels of expertise: orthopedic and trauma surgery fellows and attending surgeons) reviewed the CT scans via the Picture Archiving and Communication System (PACS) to allow dynamic analysis of the complete CT dataset with coronal, sagittal, and axial  planes.

**Fig. 2:**
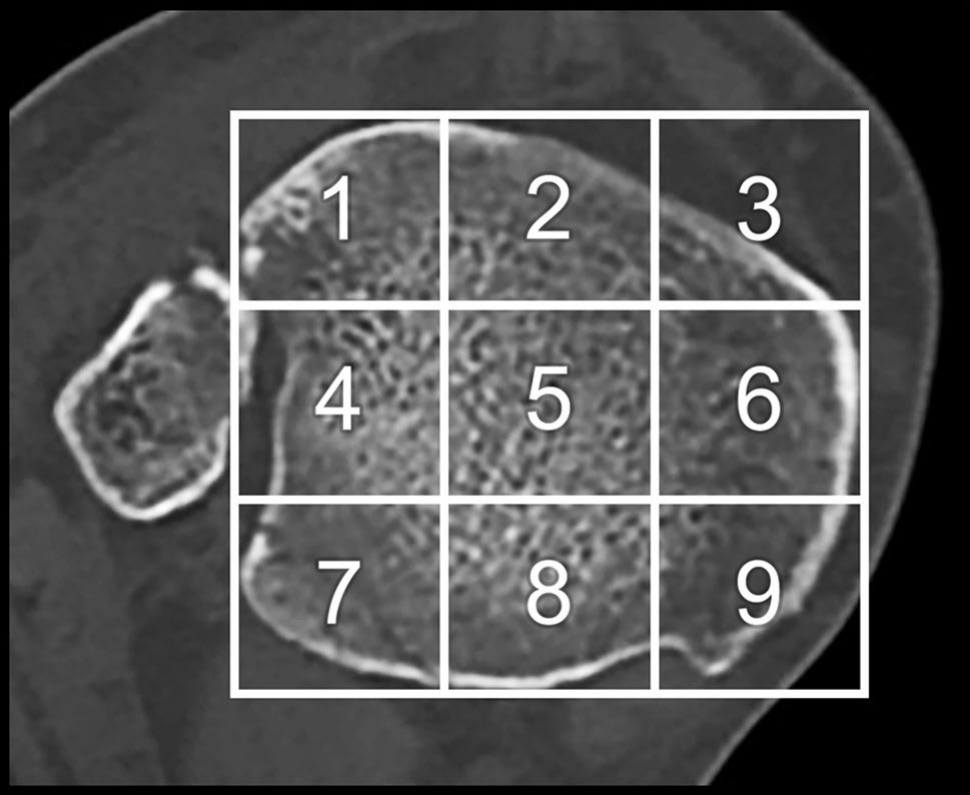
Division of the tibial plafond into nine equal parts to determine the location of the ICF

The axial plane of the CT scan was divided into nine equal parts to determine the location of the ICF. The lateral line was placed at the lateral tips of the incisura, connecting the anterior and posterior distal tibia. The medial line was placed parallel to the lateral line at the most medial part of the distal tibia. Two equally divided parallel lines between the lateral and medial lines created three equal anterior–posterior (AP) divisions. The anterior and posterior lines were created by placing two lines perpendicular to the medial and lateral lines at the anterior and posterior tips of the distal tibia. Two equally divided parallel lines between the anterior and posterior lines created three equal lateral-medial (LM) divisions. The nine regions created by the AP and LM lines were numbered from anterolateral to posteromedial according to Elias et al. and Kleinertz et al. [24, 25] (Fig. 2).

The morphology was further assessed by measuring the maximum length and height in the sagittal and width in the axial CT planes (Fig. 1). An approximate ICF volume was calculated from those values.

### Injury classification

Fracture classification and analysis of the ICFs were conducted with preoperative CT scans by all four observers. All PM fractures were classified according to Haraguchi, Bartoníček/Rammelt, and Mason (Table 1).

**Table 1:** Classifications of PM fractures according to Bartoníček/Rammelt et al. [20], Haraguchi et al. [19], and Mason et al. [21]

Bartoníček/Rammelt et al. [20]	Type 1	Extra-incisural fragment with an intact fibular notch
	Type 2	posterolateral fragment extending into the fibular notch
	Type 3	posteromedial two-part fragment involving the medial malleolus
	Type 4	large posterolateral triangular fragment (involving more than one-third of the notch)
	Type 5	irregular, osteoporotic fragments
Haraguchi et al. [19]	Type I	posterolateral-oblique wedge-shaped fragment involving posterolateral corner of tibial plafond
	Type II	transverse medial-extension fracture line extending from the fibular notch of the tibia to the medial malleolus
	Type III	one or more small shell-shaped fragments of the posterior lip of the tibial plafond
Mason et al. [21]	Type 1	small extra-articular fragment
	Type 2	fragment of the posterolateral triangle of the tibia (Volkmann area)
	Type 2A	primary fragment of the posterolateral triangle of the tibia (Volkmann area) extending into the incisura
	Type 2B	secondary fragment on the posteromedial aspect of the tibia
	Type 3	coronal plane fracture line involving the whole posterior plafond

### Statistical analysis

Normality testing was performed with the Shapiro–Wilk test. The Mann–Whitney test was used to compare ranked variables with non-normal distributions. Binominal variables from unpaired groups were compared using the Chi-square test. The level of significance for all tests was set to *p* ≤ 0.05. All data were analyzed using IBM SPSS Statistics version 28.0 (IBM, Armonk, NY).

## Results

A total of 135 patients were included in the study. The mean age was 54.4 ± 18.9 years. A total of 91 (67%) patients were female and 44 (33%) were male. Of the 135 patients, 56 (41%) were initially stabilized with an external fixator due to ankle instability before definitive surgery. All patients received primary or secondary definitive operative treatment with open reduction and internal fixation (ORIF). Overall, 76 (56%) PM fractures received surgical fixation.

### Epidemiology of the intercalary fragment

An ICF was seen in 55 of the 135 (41%) patients with a PM fracture. Patients with an ICF were significantly younger (49.4 ± 16.5 years) than the 80 patients without (57.9 ± 19.8 years, *p* = 0.007). The gender distribution was equal, with females making up two thirds of both groups. Less than half of the patients from both groups initially received external fixation due to ankle instability on the day of the injury (*p* = 0.462). Syndesmotic instability requiring tibiofibular screw fixation was seen in 82% of patients with an ICF and 83% without (*p* = 0.948). The PM fracture was surgically fixated in 82% (45 patients) of cases with an ICF during definitive operative treatment via open reduction and internal fixation, significantly more than in patients without an ICF (39%, 31 patients, *p* < 0.001) (Table 2.).

**Table 2:** Epidemiology and means of treatment for patients with posterior malleolar fractures with and without the presence of an intercalary fragment

	Intercalary fragment *n* (%)	No intercalary fragment *n* (%)	*p* value	Total *n* (%)
Total	55 (41)	80 (59)		135 (100)
Age*	49.4 ± 16.5	57.9 (SD ± 19.8)	*p* = 0.007	54.4 ± 18.9
Female	38 (69)	53 (66)	ns	91 (67)
Operative treatment	55 (100)	80 (100)	ns	135 (100)
External fixation prior to definitive treatment	25 (45)	31(39)	ns	56 (41)
Tibiofibular screw	45 (82)	66 (83)	ns	11 (82)
Surgical fixation of the posterior malleolus	45 (82)	31 (39)	*p* < 0.001	76 (56)

Statistical analysis was performed using the Mann–Whitney test for age and the Chi-square test for the others

*ns* not significant

PMFs with an ICF were accessed via a posterolateral approach in 71% of cases, while those without an ICF only utilized this approach in 26% of cases (*p* < 0.001) (Table 3). Eight percent of fractures with an ICF were fixated with a posterior screw and/or plate from posterior (*n* = 44); however, only 29% of fractures without an ICF were fixated via dorsal osteosynthesis (*n* = 23, *p* < 0.001). Detailed information about the surgical treatment of both groups is demonstrated in Table 3.

**Table 3:** Surgical treatment details for patients with posterior malleolar fractures (PMF) with and without the presence of an intercalary fragment

	Intercalary fragment *n* = 55 (%)	No intercalary fragment *n* = 80 (%)	*p* value	Total *n* = 135 (%)
PMF approach				
Percutaneous	5 (9)	9 (11)	ns	14 (10)
Posterolateral	39 (71)	21 (26)	*p* < 0.001	60 (44)
Posteromedial	2 (4)	3 (4)	ns	5 (4)
Further approaches				
Lateral	15 (27)	61 (76)	*p* < 0.001	76 (56)
Medial	45 (82)	62 (78)	ns	107 (79)
Operative technique				
AP screw	1 (2)	8 (10)	ns	9 (7)
PA screw	39 (71)	23 (29)	*p* < 0.001	62 (46)
Dorsal plate	11 (20)	1 (1)	*p* < 0.001	12 (9)

Multiple approaches and operative techniques (e.g., combination of a dorsal screw and dorsal plate) per case were possible (*ap* anterior–posterior, *pa* posterior–anterior). Statistical analysis was performed using the ChBi-square test (*ns* not significant)

**Fig. 3:**
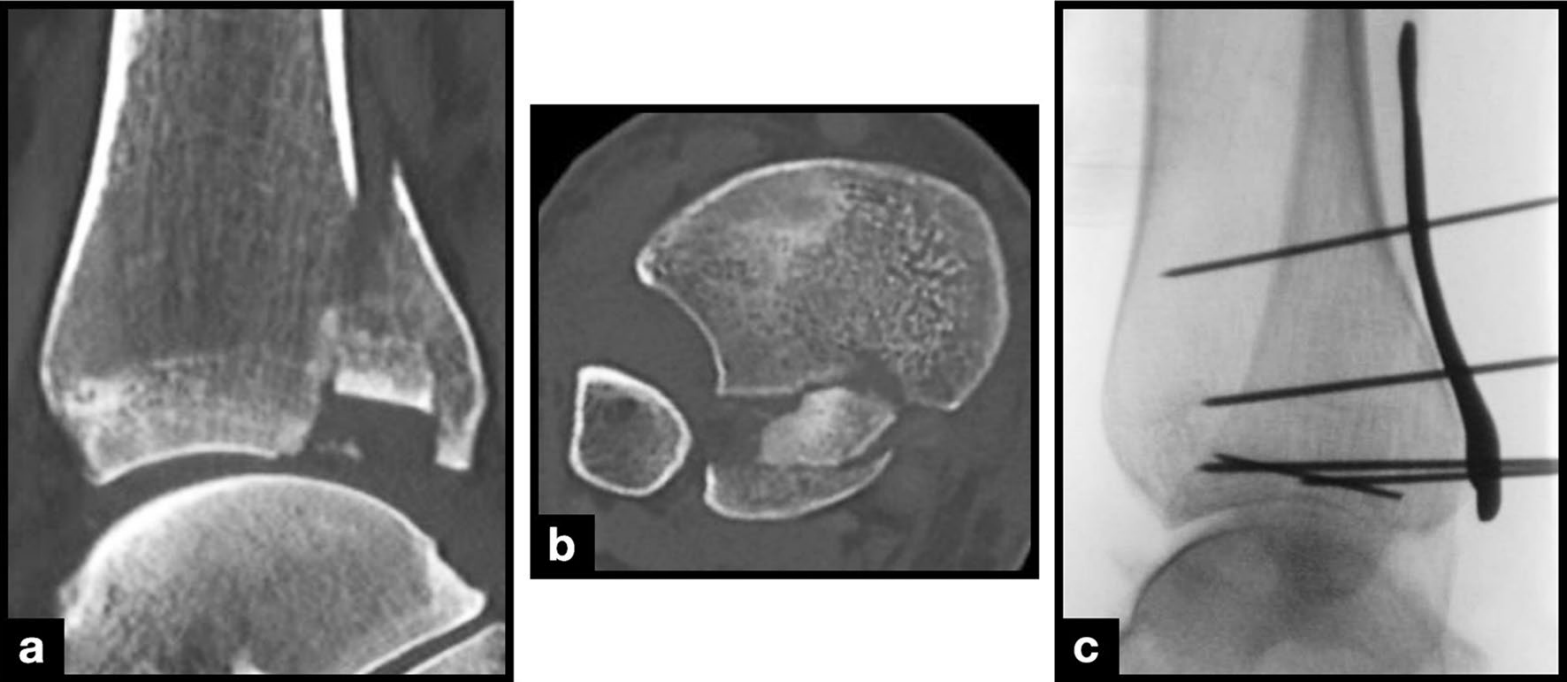
A large intercalary fragment in the **a** sagittal and **b** axial plans. **c** A lateral intraoperative X-ray with fixation of the ICF with a (lost) Kirschner wire (K-wire) and temporary plate fixation with K-wires

The ICF itself had to be directly addressed in 84% of cases (*n* = 46), of which the ICF had to be removed in 39% (*n* = 18) in cases as they interfered with the anatomic reduction of the PM fragment. Sixty-one percent (*n* = 28) of the ICFs addressed surgically were included in the osteosynthesis, 11% (*n* = 5) indirect via a percutaneous screw and 15% (*n* = 7) via a (lost) Kirschner wire (Fig. 3).

### Dislocation and location of the intercalary fragment

**Fig. 4:**
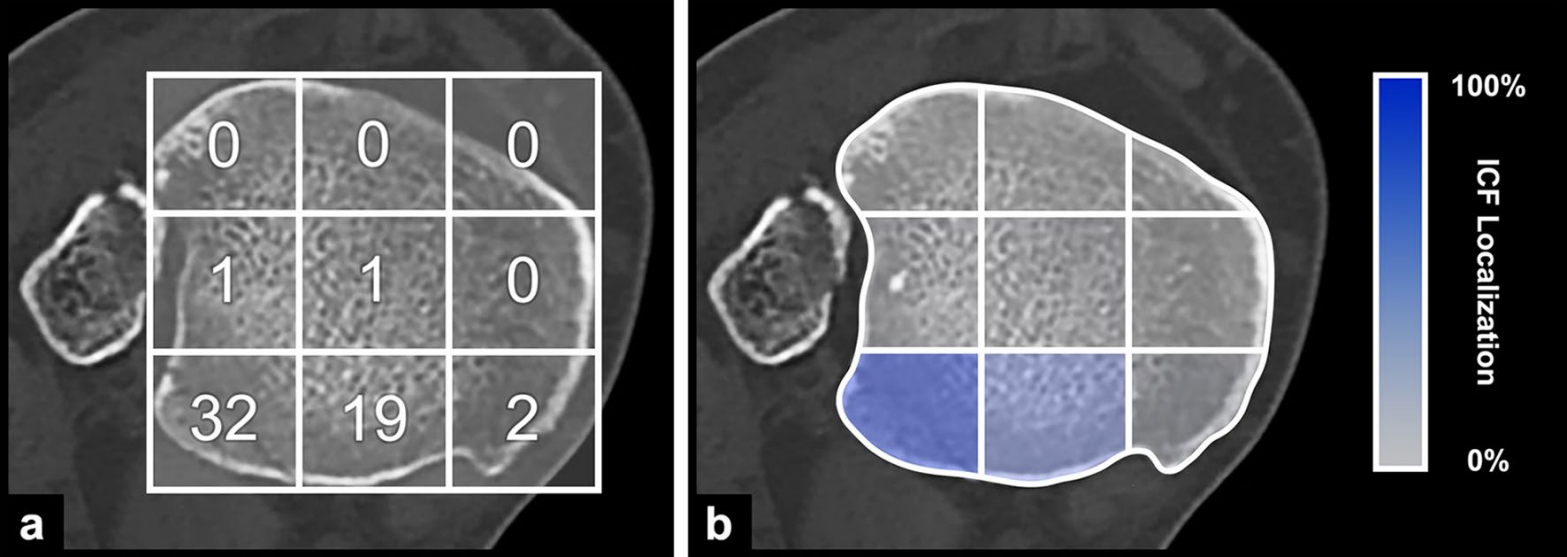
Location of the intercalary fragments within the nine regions of the CT scan axial plane in **a** absolute numbers **b** percentages

Of the 55 ICFs, 36 consisted of multiple fragments (65%). A total of 48 (87%) ICFs were dislocated. A total of 32 (58%) ICFs were in the posterolateral region and 19 (35%) in the posterocentral (Fig. 4). The centro-lateral, centro-central, and posteromedial regions were only rarely involved.

### Morphology of the intercalary fragment

The size of the ICFs varied, with a mean length of 4.4 ± 2.4 mm, height of 5.7 ± 3.4 mm, width of 8.7 ± 4.6 mm, and volume of 259.1 ± 301.4 mm^3^.

### Frequency of intercalary fragments within CT-based posterior malleolar fracture classifications

The majority of ICFs presented in Haraguchi et al. type I (53%), Bartoníček/Rammelt et al. type 2 (49%), and Mason et al. type 2A fractures (53%) (Table 4). The distribution of ICFs correlates with the PM fracture type frequency, as the aforementioned PM fracture types were the most common for each of the classifications. When looking at the relative frequencies of ICFs within each fracture type, Bartoníček/Rammelt type 3 fractures showed the highest relative presence of ICFs with 68%, followed by type 2 fractures with 41% (Table 5).

**Table 4:** Presence of an intercalary fragment in posterior malleolar fractures according to the classifications by Haraguchi, Bartoníček/Rammelt, and Mason (type 2 = Mason 2A, type 3 = Mason 2B, type 4 = Mason 3)

	Haraguchi [19]	Bartoníček/Rammelt [20]	Mason [21]
Type 1	29 (53%)	5 (9%)	5 (9%)
Type 2	21 (38%)	27 (49%)	29 (53%)
Type 3	5 (9%)	21 (38%)	19 (34%)
Type 4	n.a	2 (4%)	2 (4%)
Total	55	55	55

**Table 5:** Absolute and relative presence of ICFs within the Bartoníček/Rammelt (BR) classification

	ICF	Total	% of Total
BR 1	5	31	16.1
BR 2	27	66	40.9
BR 3	21	31	67.7
BR 4	2	7	28.6
Total	55	135	

## Discussion

Our study revealed a high frequency of ICFs in PM fractures. Compared to the cases without ICFs, the likelihood of direct open internal fixation of PM fragments during definitive operative treatment was significantly higher, as well as the need for a posterolateral approach. The majority of ICFs were located in the posterolateral and posterocentral regions. Within the CT-based PM fracture classifications, ICFs were predominately found in the typical posterolateral triangular fracture type (Haraguchi I, Bartoníček / Rammelt 2, Mason 2A).

In line with previous studies, we found a high proportion (41%) of PM fractures to be associated with the presence of an ICF. Sultan et al. reported an ICF in 43% (106 of 247) of cases [22] and Xie et al. even in 69% (75 of 108 cases) [26]. The age and gender of cases with an ICF in previous studies were in accordance with our cohort of PM fracture patients. Interestingly, patients with an ICF were significantly younger than those without. This could be due to a higher percentage of high-energy trauma in younger patients and therefore more complex fractures. Corresponding to this observation, almost all ICFs were dislocated, and the majority were multifragmentary.

Almost twice as many PM fractures with an ICF were surgically fixated compared to those without. This reflects more recent treatment recommendations [8, 10] and implicates a change in treatment if an ICF is present. The reduction of a dislocated ICF is required for the anatomic reconstruction of the distal tibial articular surface (plafond) and therefore contributes to the prevention of post-traumatic arthritis [12, 27]. If anatomic reduction of the posterior malleolar fragment is not possible, small or comminuted ICFs should rather be discarded [10]. Xie et al. showed a higher risk of articular malreduction in patients with an ICF than without [12]. Bartoníček et al. recommended a generous surgical indication for the anatomic reduction and fixation of PM fractures in the presence of an impacted ICF [23].

Similar to the results of Sultan et al. and Martin et al., the majority of ICFs in our study were located in the posterolateral (58%) and posterocentral (35%) regions [22, 28]. Xie et al. divided the tibial plafond in two parts (medial and lateral) and detected the majority (57%) of ICFs in the lateral region [26]. This leads to the presumption that a posterolateral approach should be the first choice in most cases with an ICF, which agrees with our data. Nevertheless, the final decision about the approach and method of reduction should be made based on the preoperative CT scan and soft tissue status. As expected, the ICF size varied considerably, which underlines the necessity of a preoperative CT scan to not overlook especially small ICFs. Previous studies already showed conventional radiography to be insufficient when not just assessing the extent of the fracture, but also comminution and degree of joint involvement [10, 20, 29–31].

The absolute amount of ICFs within the three established CT-based classifications by Haraguchi, Bartoníček/Rammelt, and Mason mirrored the frequency of the PM fracture types (Haraguchi type I, Bartoníček/Rammelt type 2, Mason type 2A). These findings are in accordance with Bartoníček/Rammelt, who describe an ICF in 34% of their type 2 cases [20]. Regarding the relative ICF frequency within the Bartoníček/Rammelt classification, we found the highest in type 3 fractures. Sultan et al. detected significantly more ICFs in Bartoníček/Rammelt type 3 fractures than in the other types, not differentiating between the absolute and relative frequencies. The authors speculated Bartoníček/Rammelt type 3 fractures to be posterior pilon variants, implicating rather axial than rotational forces on the distal tibial plafond [22]. We agree with Xie et al., who assume that a combination of rotation and impaction are important factors leading to an ICF in PM fractures [26].

In the clinical application, creating an extended PM fracture classification with “I = ICF” and “0 = no ICF” would be an easy solution. But when it comes to treatment recommendations, this retrospective study cannot answer if an ICF is an indication for surgery and which approach should be chosen. This remains an individual case-by-case decision which takes the patient’s age, overall condition, bone quality, and preoperative CT scan into account.

### Limitations

Even though this study was carefully designed and meticulously analyzed, it has certain limitations. Our study only includes patients with operatively treated ankle fractures, indicating a selection bias towards more severe ankle fractures. However, this seems inevitable, since PM fractures are primarily found in more complex ankle fractures [11, 23]. Further, the study was conducted with a retrospective cohort, although we included all operatively treated ankle fractures at our institution in the mentioned timespan. The ICF volume calculation is not completely mathematically correct, but indicates an estimate and demonstrates their variation in size. This study did not analyze the outcomes of patients with an ICF. For this purpose, further studies with a different protocol are required.

## Conclusion

ICFs are common in PM fractures and their size, degree of dislocation, and location are relevant in operative treatment planning. If a PM fracture is suspected, a preoperative CT scan should be added to the diagnostic protocol to detect small ICFs and plan the appropriate operative approach, reduction, and fixation, if required. Due to their location, most ICFs should be accessed using a posterolateral approach. ICFs play an important role in PM fracture treatment and an extension of the existing PM fracture classifications should be considered.
